# Readers' Questions with Practical Answers

**Published:** 1912-11-23

**Authors:** 


### INSTITUTIONAL HOUSEKEEPING.

### Readers' Questions with Practical Answers.

**Christmas Pudding for Fifty.**—We are very pleased to give you the recipes you ask for as under, and are sorry they were crowded out last week. So glad you find the Housekeeping Queries helpful. You will find the following an excellent recipe for your Christmas pudding : 2 lb. each of moist sugar, currants, raisins, and sultanas; 1 lb. each of flour and breadcrumbs and mixed candied peel; 2 lb. suet; ½ lb. almonds; 10 eggs; about one pint of milk; grated rind of three lemons; one teaspoonful of salt; one nutmeg grated; two wineglassfuls of brandy. Mix all the dry ingredients together, add the eggs and brandy and milk to bring it to the right consistency. The success of all these rich puddings depends on their thorough mixing. The probable cost will be 7s. 6d.—Emma S.

**An Excellent Mince-pie.**—For the mince-meat take equal quantities (for sixty mince-pies ½ lb. of each will be sufficient) of currants, raisins, cooking apples, beef suet, tender roast beef, and candied peel, and twelve bitter almonds. Chop these all together, add one small saltspoonful of salt, 1 oz. ground cinnamon, one saltspoonful of grated nutmeg, and the juice of one lemon, one wineglassful of cooking sherry and one of brandy. The beef and sherry can be omitted. Probable cost, including pastry, 3s. 6d.—Emma S.

**Christmas Cake.**—I have used both these recipes with success, and probably one or the other will meet your requirements. No. 1, rich cake for staff tea. Required : 1½ lb. flour, 1 lb. sultanas, 2 lb. currants, 6 oz. candied peel, which must be chopped very fine, 11 eggs, 1¼ lb. of butter, 1¼ lb. of sugar, teaspoonful of baking-powder. Cream the butter and sugar, add the eggs worked in separately, then the rest of the ingredients, which must be well mixed. Bake in a slow oven for four hours. Line the tin with greased paper before putting in the mixture. Probable cost about 5s. No. 2, not so rich : 1½ lb. flour, ½ lb. butter, 10 oz. sugar, ½ lb. raisins, 1 lb. currants, ¼ lb. candied peel, 3 eggs, two gills of milk, two teaspoonfuls of baking-powder, half-teaspoonful of salt. Rub the butter into the flour, add the other ingredients, and mix up with the eggs and milk. A little burnt sugar (gravy colouring) may be added to this to give it a rich dark colour. Bake in a moderate oven for one hour. It is always best to test cakes of this sort with a straw or knitting-needle, as an hour may not be long enough. Probable cost about 2s. 6d. If icing only is added the cost will be about 1s. more; if almond icing, about 2s. 4d. to 2s. 6d. extra.—Emma S.

**Piccalilli.**—We are pleased to furnish the recipe you require. Almost any kind of vegetable can be utilised for preparing piccalilli and the more variety the better, but it is a little late in the season to get some kinds. French beans, cauliflower, green tomatoes, white hearts of cabbage, little onions, radishes, etc., are among the ingredients. Wash all thoroughly, slice into pieces of about the same size. Drop them into a pot of very strong boiling brine and leave them to boil one minute. Then drain off, and dry in the sun. After this pour over them a good pickle, hot but not boiling; let it all stand till it is cold and then bottle and store, taking care that the pickle entirely covers the vegetables. For making the pickle the following is a good recipe : One quart of vinegar, 1 oz. of powdered ginger, ½ oz. of white pepper, ½ oz. of all-spice, 1 oz. of curry powder. Boil all together for five minutes. A little mustard added in a smooth paste while cooling improves the flavour. Thanks for your kind appreciation of this page.—Cook.

**Home-Made Essences.**—We understand your wail over expensive flavouring. There is no doubt that if you want to be very economical you must make this kind of thing for yourself. Put the peel of oranges or lemons, with the white pith scraped off, into a covered bottle or jar with some good French brandy and store it away. This makes a very fine flavouring for puddings, cakes, etc., and a little goes a long way. Tangerine oranges are particularly good for this, but do not break the oil cells. Empty pickle bottles with a spring cork can be used.

**To Utilise Cold Fish.**—There are several ways of doing this, and the following is one of the simplest : Take some scallop-shells, or the glass-shells used as butter dishes for small tables (these can be got from 1s. to 2s. a dozen); arrange some finely-sliced cucumber round the shell, leaving a space in the centre, fill it up with nice pieces of cold fish and cover with a mayonnaise sauce. Each scallop sufficient for one person. Write to us again if you want other recipes.—Sister Dora.

